# Excellent functional outcomes in patients aged 40 years or older undergoing isolated rotator cuff repair for rotator cuff tears after primary traumatic anteroinferior shoulder dislocation

**DOI:** 10.1007/s00402-025-05785-0

**Published:** 2025-04-09

**Authors:** Felix Hochberger, Marco-Christopher Rupp, Felix Boenke, Bastian Scheiderer, Sebastian Siebenlist, Lukas N. Muench, Daniel P. Berthold

**Affiliations:** 1 Department of Sports Orthopaedics, Technical University of Munich, Ismaninger Str. 22, 81675 Munich, Germany; 2 Department of Orthopaedic Surgery, Julius-Maximilians University Wuerzburg, Koenig- Ludwig-Haus, Brettreichstrasse 11, 97074 Wuerzburg, Germany; 3 Orthocenter Munich, Maximilianstraße 10, 80539 Munich, Germany

**Keywords:** Shoulder dislocation, Glenohumeral dislocation, Rotator cuff tear, Rotator cuff reconstruction, Shoulder instability

## Abstract

**Purpose:**

To investigate the functional outcomes of patients over 40 years of age who underwent isolated rotator cuff (RC) repair (RCR) for full-thickness RC tears resulting from a primary traumatic anteroinferior shoulder dislocation and to compare these outcomes with a control group of patients who underwent RCR for instability-independent RC tears, with a minimum follow-up of two years.

**Materials and methods:**

Patients aged 40 years and older were included for RCR following primary traumatic anteroinferior shoulder dislocation between 01/2012 and 06/2020 with a minimum follow-up of two years. Patients were excluded if they received an additional labral repair or capsular shift. Outcomes were compared to a control group of patients who underwent RCR without history of previous dislocations. Primary outcome measures included passive range of motion (ROM) as well as patient reported outcomes comprising the Western Ontario Shoulder Instability Index (WOSI) and Rowe score. Rates of re-dislocation were evaluated as secondary outcomes.

**Results:**

Thirty-six patients were enrolled and divided into 2 groups (*n* = 18, respectively). Demographic characteristics did not significantly differ (*p* > 0.05). At final follow-up, patients affected by instability-related RC tears showed comparable functional outcomes in terms of WOSI (427.2 ± 238.9_instability group (IG)_ vs. 431.1 ± 252.1_control group (CG)_; *p* = 0.962) and Rowe (87.5 ± 12.0_IG_ vs. 91.1 ± 10.2_CG_; *p* = 0.339) scores as well as in terms of passive ROM (abduction: 88.1 ± 4.6°_IG_ vs. 86.7 ± 11.5°_CG_; *p* = 0.637, forward elevation: 87.8 ± 6.2°_IG_ vs. 88.3 ± 5.1°_CG_; *p* = 0.772, external rotation: 55.3 ± 10.5°_IG_ vs. 50.8 ± 15.3°_CG_; *p* = 0.312, internal rotation: 65.3 ± 8.5_IG_ vs. 68.8 ± 4.9_CG_, *p* = 0.388). No patient experienced a re-dislocation.

**Conclusion:**

Patients ≥ 40 years who underwent isolated RCR without labral repair or capsular shift for a concurrent RC tear after experiencing a primary traumatic anteroinferior shoulder dislocation, achieved favorable functional outcomes along with absence of re-dislocations.

**Study design:**

Retrospective case series; Level of Evidence IV.

## Introduction

There is a substantial body of evidence demonstrating that patients over 40 years of age have an elevated risk of sustaining a rotator cuff (RC) injury as well as fractures of the glenoid or humerus compared to the younger patient population in the setting of a traumatic shoulder dislocation [2, 11, 12, 23, 26, 28–30, 33, 34]. More specifically, it has been shown that in this subgroup of patients the likelihood of full-thickness RC tears increases with age, while the probability of a Bankart lesion decreases [35].

If left undiagnosed or improperly treated, a rotator cuff injury in the setting of a primary instability event can cause substantial functional impairment, which may ultimately result in loss of range of motion, pain, and onset of cuff tear arthropathy [11, 32, 35]. Given these reasons, the surgical rationale for performing a RCR is strong in this patient population, while there remains ambiguity among shoulder surgeons on whether to perform a concomitant repair of the capsulolabral complex [22, 30]. While this procedure is commonly recommended for younger patients [17], it may not be advisable for older patients, as it may result in greater soft tissue-mediated stiffness of the glenohumeral joint with limited glenohumeral range of motion occurring over time [35]. Furthermore, a subsequent overconstraint of the glenohumeral joint may lead to an accelerated degeneration of the cartilage in elderly patients [35]. Lastly, given that the re-dislocation rate is substantially reduced compared to younger patients [17], a concomitant stabilization procedure may be a surgical overtreatment for these patients. Consequently, there is currently no established “gold standard” for determining which surgical treatment is necessary [11], and the decision may depend on factors such as the nature of the injury and the patient’s and surgeon’s personal preference.

To date, it remains unclear whether patients undergoing an isolated RCR for a primary anterior shoulder dislocation without labral repair or capsular shift with a concurrent RC tear experience a different outcome compared to patients undergoing a RCR for instability-independent RC tears.

Thus, the purpose of this study was to investigate functional outcomes in patients > 40 years of age who underwent isolated RCR following primary traumatic anteroinferior shoulder dislocation and compare the outcomes to a control group of isolated RCR for instability-independent RC tears. The primary hypothesis was that satisfactory outcomes would be achieved and that there would be no significant differences in functional outcomes compared to a control group of patients who underwent RCR for instability-independent RC tears. The secondary hypothesis was that there would be a low rate of re-dislocations at a minimum follow-up of two years.

## Materials and methods

### Study design

Institutional review board permission was obtained prior to initiation of the study (43/22 S-KK). A retrospective comparative study with a minimum follow-up of 2 years was conducted at the author´s institution, involving 2 groups of patients between January 2012 and June 2020: (1) patients who underwent arthroscopic RCR following primary traumatic anterior shoulder dislocation with a resulting lesion of the capsulolabral complex and a concomitant RC tear, who were aged 40 years and older; (2) a comparative patient cohort who underwent isolated instability-independent arthroscopic RCR without a prior history of shoulder dislocation and/or instability during the same period of time. Patients were excluded if they had a previous history of glenohumeral instability and/or (capsulo)-ligamentous and/or bone-based stabilization procedures, concomitant fractures of the humeral head or glenoid requiring surgery, neurovascular injuries, and an age < 40 or > 75 years. The selection of patients in the control group was performed based on the epidemiological characteristics in the instability group such as sex, age, and BMI.

### Surgical procedure and postoperative care

In all cases, a diagnostic arthroscopy of the glenohumeral joint was performed [9]. The anterior labrum was evaluated and a Bankart lesion was diagnosed. Labral repair or capsular shift was not performed. Reconstruction of the supraspinatus tendon (SSP) was performed using the double-row technique [3]. Reconstruction of the subscapularis tendon (SSC) was performed using either the single- or double-row technique, depending on the tear size [21]. Depending on the intraoperative evaluation, either tenotomy or tenodesis of the long biceps tendon (LHBT) was performed in an open subpectoral technique using all suture anchors [13, 20].

Postoperative rehabilitation consisted of wearing a specific shoulder brace in 15° abduction (SAS comfort, Fa. Medi, Bayreuth, Germany) for 6 weeks. The restriction of passive range of motion in terms of postoperative rehabilitation was related to the type of rotator cuff tendon that had been reconstructed. In case of isolated SSP repair during the first 3 weeks, passive abduction/adduction and flexion up to 90° were allowed and internal and external rotation were free. If SSC reconstruction was additionally performed, passive external rotation for the first 3 weeks was suspended. Active-assisted glenohumeral range of motion (abduction and adduction as well as flexion up to 90°, internal and external rotation free) was started from the fourth postoperative week. In case of SSC reconstruction, active-assisted external rotation starting from week 7 after surgery. Transition to free active glenohumeral range of motion starting in the seventh week. In case of additional LHBT tenodesis, no active biceps exercises for 6 weeks were allowed. During the first 6 weeks, training for scapulothoracic rhythm and stabilizing exercises were recommended. Weight bearing training was started within 3 to 5 months.

### Outcome measures

#### Primary outcome

The Western Ontario Shoulder Instability Index (WOSI), Rowe Score, glenohumeral range of motion (ROM), comprising passive forward elevation (FE), passive abduction (ABD), passive external rotation (ER) and passive internal rotation (IR), were assessed at final follow-up. Furthermore, baseline demographic factors such as age, sex, body mass index (BMI), and ASA status (ASA - American Society of Anesthesiologists) were evaluated through chart review.

#### Secondary outcome

Rates of re-dislocation were evaluated as secondary objectives at final follow-up.

### Statistical analysis

A power analysis was performed to determine the capability of the sample size to detect a clinically meaningful difference of 9.7 points in Rowe score [27]. Assuming a standard deviation of 10 points and an allocation ratio of N2/N1 of 1, a total sample size of 28 patients would provide 80% power at an alpha-level of 0.05 determined in an a priori power analysis, performed with G*Power (Erdfelder, Faul, Buchner, Lang, HHU Düsseldorf, Düsseldorf, Germany) [10].

Continuous variables were reported as mean ± standard deviation and categorical variables were reported as counts and percentages. The Shapiro-Wilk test was used to evaluate the distribution of continuous variables. Depending on the respective distribution of the data, parametric tests (unpaired t-test) or non-parametric tests (Mann-Whitney U test) were used to compare continuous variables between groups. To compare pre- and postoperative values, the non-parametric Wilcoxon test for two related samples or parametric paired t-test was utilized. Categorical variables were compared using binary Fisher’s exact tests or Chi-square test as appropriate. Confidence intervals of 95% were provided and a significance level of *p* < 0.05 was used. The statistical analysis was performed using SPSS software version 26.0 (IBM-SPSS, New York, USA).

## Results

### Patient demographics

**Fig. 1:**
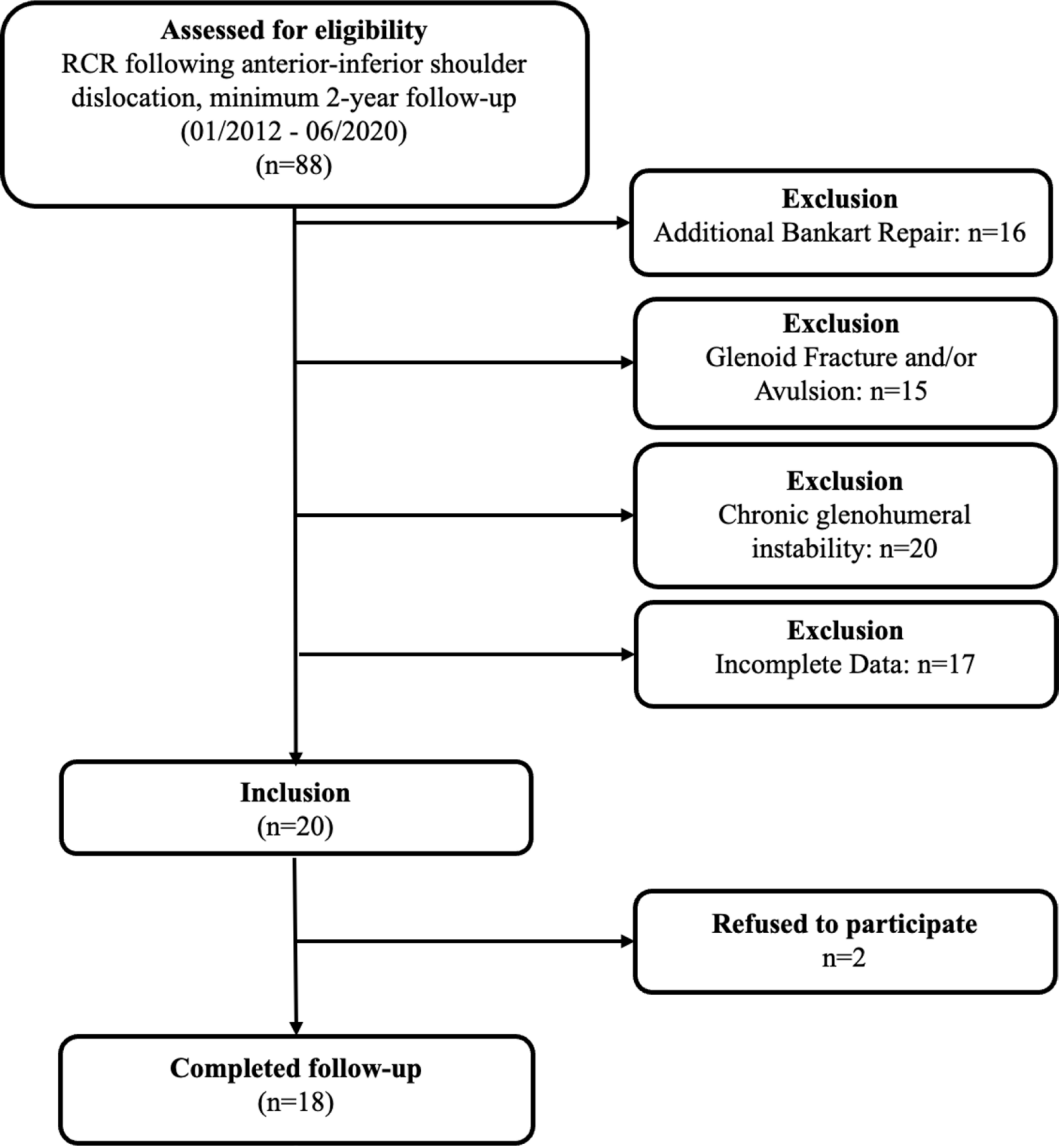
Flowchart displaying patients meeting study criteria

An analysis of the institutional database identified 88 patients who underwent RCR as a result of an antero-inferior shoulder dislocation with confirmed anteroinferior labral lesion and concomitant RC tear between January 2012 and June 2020. Fifteen patients were excluded due to the presence of an additional glenoid avulsion in terms of a Perthes lesion and/or fracture of the glenoid and 20 patients because of chronic glenohumeral instability prior to surgical intervention. Additionally, 16 patients were excluded due to an additional Bankart repair. Incomplete Data was available in 17 patients and 2 patients refused to participate in the study, leaving 18 patients for clinical evaluation (Fig. 1).

Based on the epidemiological characteristics of the instability group, 18 patients were enrolled in the control group. The mean age of the patients was 58.6 ± 9.9 years (range: 40–75) for the instability group and 58.8 ± 8.3 years (range: 45–72) for the control group. Mean follow-up was 55.3 ± 28.1 months (range: 24–109 months) in the instability group and 70.7 ± 17.2 months (range: 51–107 months) in the control group. Patient demographics are demonstrated in Table 1.

**Table 1:** Patient demographics; *Abbreviations: m = months; sd = standard deviation*

Variable	Instability Group	Control Group	*p*-value
Sex, n (%)			
Male	10 (55.6%)	11 (61.1%)	
Female	8 (44.4%)	7 (38.9%)	
Age at surgery, mean ± SD, m	58.6 ± 9.9	58.8 ± 8.3	*P* = 0.942
Follow-up, mean ± SD, m	55.3 ± 28.1	70.7 ± 17.2	*P* = 0.060

### Primary outcome measures

**Fig. 2:**
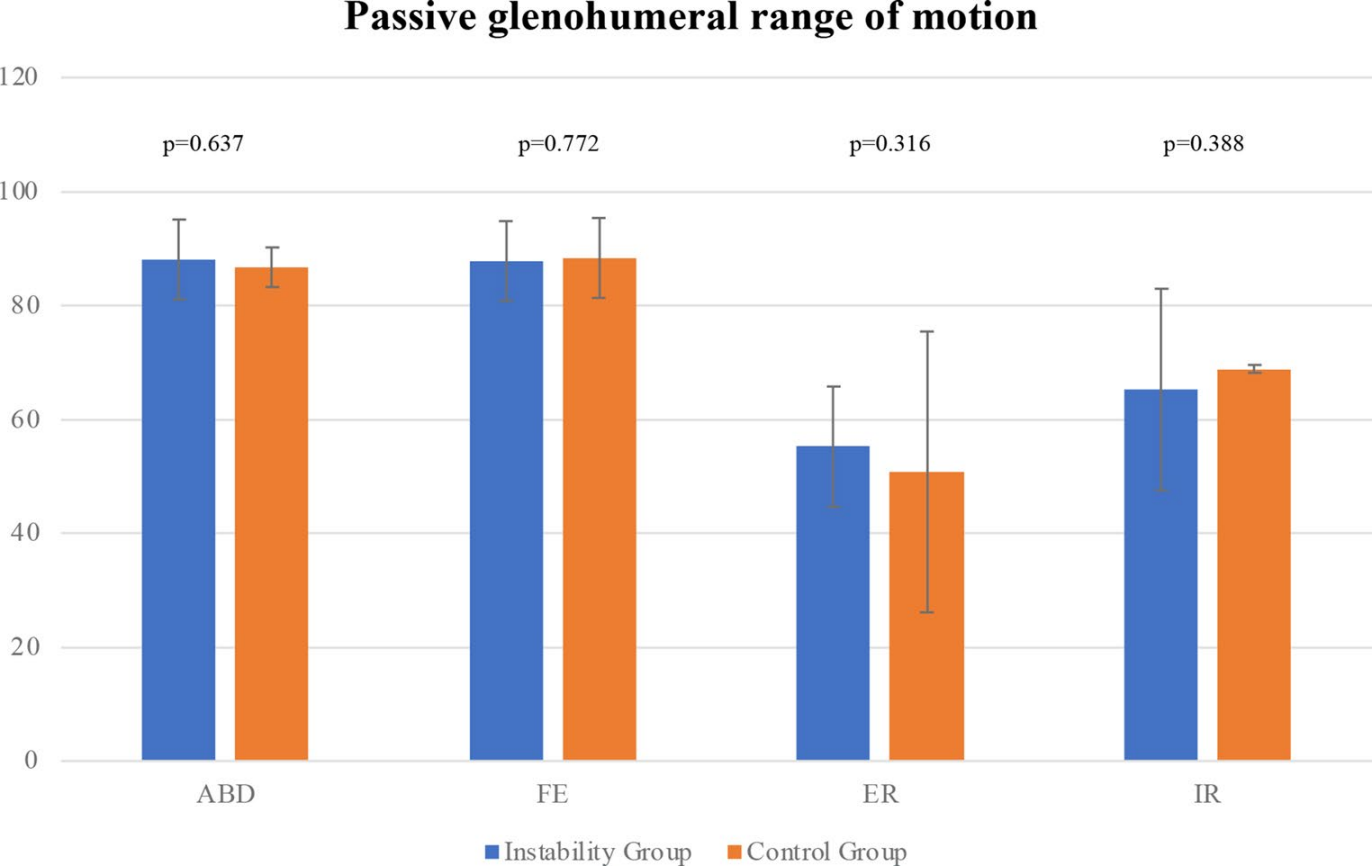
Bar chart depicting postoperative passive glenohumeral range of motion at final follow-up. *Abbreviations: ABD*,* passive abduction; FE*,* passive forward elevation; ER*,* passive external rotation; IR*,* passive internal rotation*

**Fig. 3:**
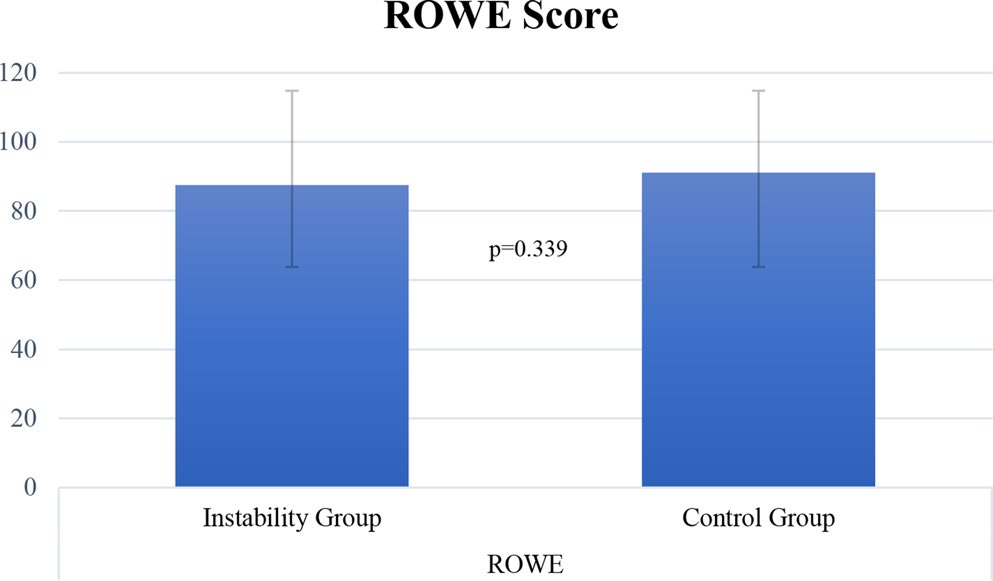
Postoperative Rowe scores for patients of both groups at final follow-up

**Fig. 4:**
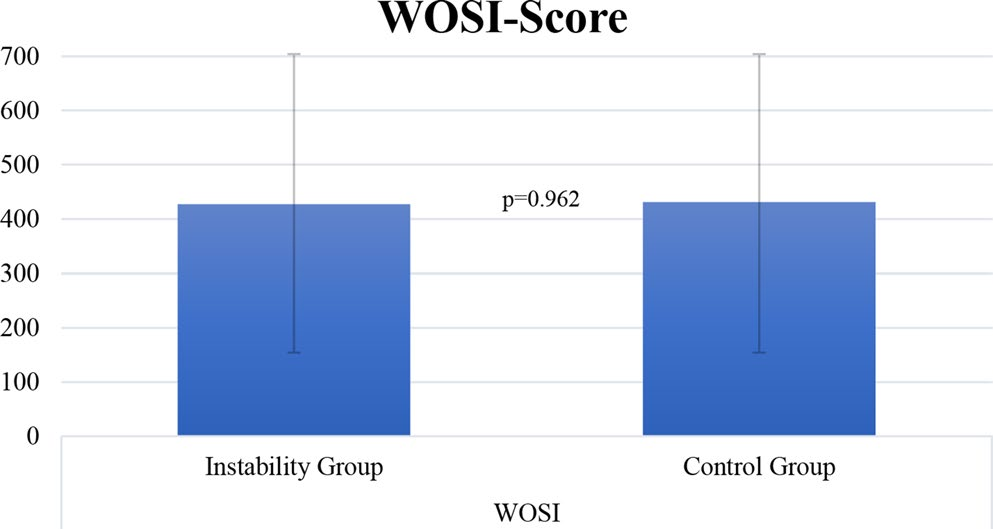
Postoperative WOSI scores for patients of both groups at final follow-up

Patients of both groups achieved similar WOSI (427.2 ± 238.9 instability group vs. 431.1 ± 252.1 control group; *p* = 0.962) and Rowe scores (87.5 ± 12 instability vs. 91.1 ± 10.2 control group; *p* = 0.339) (Table 3; Figs. 3 and 4). Additionally, no significant differences (Fig. 2 and Table 2) were found in passive glenohumeral ROM between the groups at final follow-up (*p* > 0.05, respectively).

**Table 2:** Passive glenohumeral range of motion in degree at final follow-up. Data are shown as mean ± sd unless otherwise indicated. ABD, passive abduction; FE, passive forward elevation; ER, passive external rotation; IR, passive internal rotation

Passive glenohumeral ROM			
	Instability Group	Control Group	*p*-value
ABD	88.1 ± 4.6	86.7 ± 11.5	0.637
FE	87.8 ± 6.2	88.3 ± 5.1	0.772
ER	55.3 ± 10.5	50.8 ± 15.3	0.316
IR	65.3 ± 8.5	68.8 ± 4.9	0.388

**Table 3:** Postoperative Western Ontario shoulder instability index (WOSI) and Rowe scores at final follow-up. Data is presented as mean ± standard deviation as well as range

	Instability Group	Control Group	*P*-value
Rowe	87.5 ± 12 (55–100)	91.1 ± 10.2 (65–100)	*P* = 0.339
WOSI	427.2 ± 238.9 (210–970)	431.1 ± 252.1 (210–1200)	*P* = 0.962

### Secondary outcome measures

At the final follow-up, there was no patient in the instability group who experienced a re-dislocation. Likewise, in both groups, none of the patients required revision surgery.

## Discussion

The most important finding of this study was that patients > 40 years of age who sustained a primary traumatic anteroinferior shoulder dislocation experienced favorable functional outcomes following RC repair without additional labral repair or capsular shift following at a minimum two-year follow-up. Notably, no significant differences in functional outcomes were found in comparison to a control group of patients with instability-independent RC tears. Additionally, none of the patients in the instability group experienced a recurrent dislocation throughout the follow-up period. Consequently, within this patient cohort, patients can be expected to follow the clinical trajectory of patients with RC tears independent of shoulder instability. In these patients, an additional arthroscopic Bankart repair may not be necessary and be reserved for patients with chronic symptomatic instability [38].

Numerous studies have been conducted to investigate various techniques, advantages, complications, and functional outcomes of arthroscopic labral repair [7, 16]. Subsequently, the surgical management of glenohumeral instability, particularly in younger patients, is widely supported in current literature [1]. However, limited data is available on the surgical management and clinical outcomes of RC tears following shoulder dislocation, despite their clinical significance, especially in elderly patients. To date, there remains a lack of consensus regarding the optimal approach for managing these patients– a matter of significant clinical importance. This is particularly notable given that individuals over 60 years of age may face a substantial 70% risk of experiencing a full-thickness RC tear subsequent to a traumatic shoulder dislocation [14, 35].

Consequently, the present data aimed to fill this gap of knowledge and demonstrated favorable clinical and functional results after RCR in cases of primary traumatic shoulder dislocation. These findings are consistent with a previous study by Voos et al., in which good clinical outcomes were observed in a subgroup of patients who underwent simultaneous RCR and labral repair, without any instances of re-dislocation during the follow-up period [37]. It is important to note that the authors included both acute and chronic shoulder instabilities, without distinguishing between partial and complete RC tears. These factors can potentially influence postoperative outcomes, particularly the risk of re-tears and recurrence of instability. In a recent retrospective cohort analysis conducted by Marsalli et al. [24], the recurrence of instability after RCR following primary traumatic anterior shoulder dislocation was investigated in patients over the age of 40. The study included patients with an average age of 57 years who had experienced their first traumatic anterior shoulder dislocation, had reparable RC tears, and did not undergo labral or bony Bankart lesion repair. Only 1.2% of the patients suffered a re-dislocation during follow-up, leading to revision surgery. Interestingly, the authors also reported that age, subscapularis tears, bony Bankart injuries, humeral defects, and concomitant neurologic injuries were no risk factors for recurrent instability.

Similar findings were reported by Chan et al., who performed a retrospective chart review to evaluate the functional outcomes of anterior shoulder instability repair (Bankart or bony Bankart repair) with and without RCR in patients over the age of 40 [8]. The study established four cohorts and performed a subgroup analysis. The results showed comparable functional outcomes among all four groups, suggesting that concomitant RC tears should be surgically treated, particularly in patients over the age of 40. Recurrent instabilities were not observed in their study. However, no control group was available.

Previous studies addressing shoulder dislocation have already recognized the occurrence of RC tears following glenohumeral dislocation [19, 25, 33]. The impact of RCR on the biomechanical stability of the glenohumeral joint has been extensively investigated. It has been observed that the RC plays a significant role in both active and passive stability of the glenohumeral joint [6, 18, 36]. Inadequate reconstruction of the RC can lead to reduced shoulder function, increased risk of degenerative changes, and particularly a higher likelihood of recurrent instability [4, 11, 12, 15].

Inadequate treatment or neglect of a concomitant RC tears poses a higher risk for recurrent instability events. In a recent prospective cohort study by Robinson et al., it was observed that patients who had a concomitant RC defect following their initial shoulder dislocation showed a 29.8% increased relative risk of re-dislocation [31]. The results of the present study indicate that none of the patients experienced recurrent instability after undergoing RCR. This provides additional support to these previously published biomechanical studies, underscoring the importance of an intact RC for maintaining stability of the glenohumeral joint [5]. Consequently, prolonged pain and functional impairment for a duration of up to 3 weeks subsequent to a traumatic anterior dislocation of the shoulder may necessitate additional assessment of the RC using advanced imaging modalities, including MRI or ultrasound. Prompt identification of RC pathology in these individuals is of utmost importance, as it facilitates expeditious recuperation of functionality and improves overall prognoses for these patients.

### Limitations

This study is subjected to several limitations. Firstly, the data analysis was retrospective in nature, which introduces the possibility of selection bias. Secondly, the study’s sample size was small due to narrow inclusion criteria. However, power-analysis revealed that a total sample size of 28 patients would provide enough power to support the findings. Third, it is important to note that no radiographic imaging modalities such as X-ray, ultrasound, or MRI were employed during follow-up, preventing determination of the incidence of re-tears of the RC. Nevertheless, no clinical signs of re-tear were observed. Fourth, the minimum follow-up was only 24 months, thereby potentially rendering it insufficient for a comprehensive assessment of the true occurrence rate of recurrent glenohumeral instability among these patients. Lastly, the presence of pre-existing asymptomatic RC tears prior to experiencing traumatic shoulder dislocation and its potential contribution to shoulder instability remains uncertain.

## Conclusion

Patients aged 40 years and older, who underwent isolated RCR without labral repair or capsular shift for a concurrent RC tear after experiencing a primary traumatic anteroinferior shoulder dislocation, achieved favorable functional outcomes and showed no re-dislocation. Functional outcomes were similar to those of patients who underwent RCR without previous anteroinferior shoulder dislocation.
